# Letter to the editor regarding ‘How to support researchers in the primary care setting?’

**DOI:** 10.1080/02813432.2025.2507270

**Published:** 2025-05-17

**Authors:** Filipe Prazeres

**Affiliations:** ^a^ Departamento de Ciências Médicas, Faculdade de Ciências da Saúde, Universidade da Beira Interior, Covilhã, Portugal; ^b^ USF Beira Ria, Gafanha da Nazaré, Portugal; ^c^ RISE-Health, Departamento de Medicina da Comunidade, Informação e Decisão em Saúde, Faculdade de Medicina, Universidade do Porto, Porto, Portugal

Dear Editor,

I read with interest the recently published editorial, ‘How to support researchers in the primary care setting?’ by Gyllenberg et al. [1] in the Scandinavian Journal of Primary Health Care. The editorial addresses key barriers to conducting primary care research – specifically, limited time, lack of funding, and inadequate research infrastructure – and describes how a Finnish research network, the Academic Health Center, has worked to mitigate these challenges [1].

I agree with the authors that sharing successful experiences such as this can contribute to strengthening the global primary care research community, with the ultimate goal of improving population health. As a practicing family medicine specialist in Portugal, I found the editorial particularly relevant for several reasons.

First, while research in the field of Family Medicine in Portugal has been growing, it still faces significant limitations, particularly in terms of international impact and recognition [2,3]; and the barriers are very similar to those identified by Gyllenberg et al. [1]: limited time allocated for research, insufficient (public) funding, and a lack of institutional and other research support services [4].

Second, in Portugal, were established under Decree-Law No. 61/2018, as of now, twelve Clinical Academic Centers (Centros Académicos Clínicos, CAC) to foster collaboration among hospital-based healthcare providers (including, to some extent, primary healthcare centers), academic institutions, and research units [5,6]. These centers aim to promote synergies, resource sharing, and the development of innovative programs and partnerships [5,6]. However, in my opinion, the primary focus of the CACs remains hospital-based and biomedical, with limited involvement of primary care in research activities and little scientific output addressing real-world primary healthcare settings and the everyday problems faced by patients in the community. This is what sets Portugal apart from the Finnish model described by Gyllenberg et al. [1], which is centered on primary healthcare.

Third, the Finnish model could be a good inspiration for Portugal by encouraging the creation of a national research network of medical schools with direct partnerships with Family Health Units to better organize and support primary healthcare research. Such a network could include the development of thematic research areas dedicated to primary healthcare, based on problems observed in everyday practice and supported by real-world data (e.g. from BI-CSP). Topics could include chronic disease, multimorbidity, and elderly care, among others.

Educational support is also important. The Finnish network provides seminars on practical topics [1]; in Portugal, similar training initiatives could be done in collaboration with universities, focusing on skills such as publishing in scientific journals or applying for research funding. Additionally, Finland offers peer groups and mentoring opportunities [1], which help researchers feel less isolated and facilitate knowledge exchange. In Portugal access to mentors and clinical research training have previously been identified as factors that promote research engagement [7]; improving these areas would enhance both skills and motivation.

Finally, structural changes are necessary in Portugal to ensure that family medicine specialists have protected time for research, particularly within the national health service. There is also a need for dedicated funding and leadership to empower more professionals in Portuguese primary care to engage in research and, ultimately, to improve healthcare for all. Encouraging research is essential to strengthening Family Medicine as a scientific and academic discipline in Portugal.
